# Dr. Power, of Dublin

**Published:** 1861-09

**Authors:** 


DR. POWER, OF DUBLIN.—Dr. John Hatch Power, an able surgeon and writer, was recently elected Professor of Surgery to the Royal College of Surgeons in Ireland, in the room of the late Dr. Porter.

